# Audiovestibular Quantification in Rare Third Window Disorders in Children

**DOI:** 10.3389/fneur.2020.00954

**Published:** 2020-09-16

**Authors:** Soumit Dasgupta, Sudhira Ratnayake, Rosa Crunkhorn, Javed Iqbal, Laura Strachan, Shivaram Avula

**Affiliations:** ^1^Department of Paediatric Audiology and Audiovestibular Medicine, Alder Hey Children's NHS Foundation Trust, Liverpool, United Kingdom; ^2^Department of Paediatric Radiology, Alder Hey Children's NHS Foundation Trust, Liverpool, United Kingdom

**Keywords:** third window, semicircular canal dehiscence, X linked gusher, audiovestibular, children, HRCT, vHIT, cVEMP

## Abstract

Third window disorders are structural abnormalities in the bony otic capsule that establish a connection between the middle/inner ear or the inner ear/cranial cavity. Investigated extensively in adults, they have hardly been studied in children. This study is a retrospective study of children (aged 5–17 years) diagnosed with rare third window disorders (third window disorders reported rarely or not reported in children) in a tertiary pediatric vestibular unit in the United Kingdom. It aimed to investigate audiovestibular function in these children. Final diagnosis was achieved by high resolution CT scan of the temporal bones. Of 920 children attending for audiovestibular assessment over a 42 month period, rare third windows were observed in 8 (<1%). These included posterior semicircular canal dehiscence (*n* = 3, 0.3%), posterior semicircular canal thinning (*n* = 2, 0.2%), X linked gusher (*n* = 2, 0.2%), and a combination of dilated internal auditory meatus/irregular cochlear partition/deficient facial nerve canal (*n* = 1, 0.1%). The majority of them (87.5%) demonstrated a mixed/conductive hearing loss with an air-bone gap in the presence of normal tympanometry (100%). Transient otoacoustic emissions were absent with a simultaneous cochlear pathology in 50% of the cohort. Features of disequilibrium were observed in 75% and about a third showed deranged vestibular function tests. Video head impulse test abnormalities were detected in 50% localizing to the side of the lesion. Cervical vestibular evoked myogenic potential test abnormalities were observed in all children in the cohort undergoing the test where low thresholds and high amplitudes classically found in third window disorders localized to the side of the defects in 28.5%. In the series, 71.4% also demonstrated absent responses/amplitude asymmetry, some of which did not localize to the ipsilesional side. Two children presented with typical third window symptoms. This study observes 2 new rare pediatric third window phenotypes and the presence of a cochlear hearing loss in these disorders. It emphasizes that these disorders should be considered as an etiology of hearing loss/disequilibrium in children. It also suggests that pediatric third window disorders may not present with classical third window features and are variable in their presentations/audiovestibular functions.

## Introduction

The human ear consists of 2 mobile normal windows for transmission of sound between the middle and the inner ear, namely the oval window and the round window. There are other windows called third windows that are present, and these windows connect the inner ear to the cranial cavity, for example, the cochlear aqueduct, the vestibular aqueduct, and the numerous bony channels that conduct the nerves and vessels entering or exiting the inner ear from/to the posterior cranial fossa (1). These normal third windows in normal physiological conditions are of high impedance, do not affect inner ear sound conduction, and do not influence the functional sound flow (2).

Pathological third windows, on the other hand, do interfere with transmission of the cochlear traveling wave generated at the oval window, as these windows do not offer high impedance to acoustic transmission. They shunt or deviate the acoustic energy from the middle ear, thereby leading to a drop in air conducted sound thresholds and improve the bone conduction thresholds as they provide an alternate low impedance path, bypassing the oval-round window classical low impedance pathway (1). Invariably, these third windows are due to defects in the bony otic capsule.

Regardless of the anatomical location of the pathological third window, i.e., whether it is a direct physical connection between the middle and the inner ear or between the inner ear and the cranial cavity, these disorders generate typical third window features that include conductive hearing loss, sound, or positive pressure induced dizziness (Tullio's or Hennebert's phenomenon), disequilibrium, autophony, and conductive dysacusis [magnified perception of sounds generated by the body, e.g., gaze evoked tinnitus (3)] in addition to occasional oscillopsia, phonophobia, pulsatile tinnitus, and high amplitude, low threshold vestibular evoked myogenic potentials (4). These are called third window effects; however, although observation of these symptoms constitute the diagnostic criteria, some of them may be absent, especially depending on the functional status of the audiovestibular system (5).

The first pathological third window identified was the dehiscence of the superior semicircular canal in 1998 (6). Since then, there has been plenty of research not only in this particular disorder but also in third windows in general in the adult population. A recent third window was identified by Blake et al. (7) as the cochlear-facial nerve dehiscence (CFD). Several pathological third window disorders have been identified (Table 1). The most studied third window disorder remains the superior semicircular canal dehiscence (SSCD).

**Table 1:** Identified third window disorders [after Wackym et al. (3), Scarpa et al. (8)].

1. Superior, posterior, and lateral semicircular canal dehiscence 2. Cochlea-facial nerve dehiscence 3. Cochlea-internal carotid artery dehiscence 4. Cochlea-internal auditory canal dehiscence 5. X linked gusher syndrome 6. Perilymph fistula 7. Facial nerve canal dehiscence 8. Wide vestibular aqueduct in children 9. Posttraumatic hypermobile stapes footplate 10. Otosclerosis with internal auditory canal involvement 11. Bone dyscrasias for example Paget's disease of the bone and osteogenesis imperfecta 12. Endolymphatic hydrops

Intuitively and logically, the etiology of third window disorders can be deemed developmental or traumatic (9–11) if we consider SSCD. The manifestation in SSCD may be late as the dimensions may increase with age, leading to frank symptoms if SSCD is acquired (12). Canal dehiscences may be a part of more extensive cochleovestibular dysplasias, e.g., with hypoplastic cochlear or vestibular system (13) or CDH23 mutations with Usher syndrome (14). Recently, a genetic SSCD has been proposed (15). However, it must be remembered that the prevalence of SSCD with cochleovestibular dysmorphology is the same as SSCD without any other inner ear structural abnormality. This raises the possibility that a third window structural abnormality may be a *de novo* or standalone abnormality (13).

Structural and bony otic capsule abnormalities can be proposed to possess a similar etiology, although given their rarity, evidence is yet to emerge. The commonest third window disorder in children is the enlarged vestibular aqueduct (EVA) that can accompany a fully blown systemic genetic syndrome, e.g., the CHARGE (coloboma, heart defects, atresia choanae, growth retardation, genital abnormalities, and ear abnormalities) or the BOR (branchio-oto-renal) syndrome and in 20% cases may be a feature of Pendred syndrome (16). X linked gusher is an isolated otic capsule abnormality and is caused by a mutation in POU3F4 gene (17).

The objective confirmation of a third window abnormality is by demonstrating the third window effect by vestibular evoked myogenic potential (VEMP) parameters (lowering of threshold and increase in amplitude in the affected side) and by high resolution computerized tomographic scans (HRCT) of the temporal bones with optimal cuts and special views (9). VEMPS show typical third window characteristics. The lowering of impedance of the acoustic traveling wave and the third window shunted sound energy passing through the vestibular system makes it hyper reactive to the sound (18, 19), generating these typical features. Sensitivity, and specificity to diagnose a third window abnormality is high with VEMPS (20). HRCT is the gold standard of objective confirmation although it may still over diagnose the condition even when taken in slices of <0.625 mm and in the Stenver or Poschl views (9). Another observation proposed by Wackym et al. (21) is that there may be negative CT scans with typical symptoms which are responsive to surgery for third window disorders.

Only EVA as a third window disorder has been studied extensively in children as it is relatively common. In a large series comprising 221 children with mainly sensorineural hearing loss, 8.6% demonstrated an isolated EVA whilst 3.16% showed an EVA that was associated with other inner ear anomalies (22). Gopen et al. (16) in a review article observed that 20–100% of children with EVA may present with a vestibular symptom, and they invariably present with hearing losses.

Other third window disorders are rare in children including canal dehiscences (13). There are limited studies investigating SSCD in children. Dasgupta and Ratnayake (5) pointed out that SSCD in children might not present with classical third window features as they may not be able to describe these symptoms or because the defects might not have attained the dimensions to cause an overt third window symptom. Other researchers have reached similar conslusions (23, 24). In other words, SSCD in children might not generate the classical SSCD syndrome found in adults that by definition is a constellation of clinical symptoms and audiovestibular tests. SSCD has been reported in the case series by Chen et al. (25) who also reported posterior semicircular dehiscences (PSCD) in a cohort of 113 presenting with hearing loss with a 15% prevalence and by Lee et al. (23) who observed that hearing loss and disequilibrium were the commonest presenting features. Meicklejohn et al. (26) in live and cadaveric temporal bone dissections detected that prevalence of radiologic semicircular canal dehiscences declined with increasing age, reinforcing the idea that otic capsule thickens with age. He also observed normal, mixed, and sensorineural hearing losses in his cohort. A 6.2% incidence of SSCD was found in a large multicentre review by Sugihara et al. (27). Near dehiscences or where the semicircular bone is thinned but not frankly dehiscent can generate third window features and respond to third window surgery (28). They are rare and have not been investigated in detail.

There have been isolated case reports and series reports regarding X linked gushers (29). CFD has been reported only in 7 children (3, 30) and after an extensive search of literature, these authors were unable to find any child being reported with any other rare third window disorders, e.g., the carotid artery-cochlear dehiscence (CACD) that is very rare in adults as well (31).

The present study is a retrospective study investigating these rare third window disorders in children. This study reports subjective and objective audiovestibular quantification in a group of children with different but hardly reported or not reported third window structural disorders. This is the first time that we are reporting objective vestibular quantification in some groups of these children from a tertiary pediatric balance unit in the United Kingdom.

## Patients and Methods

### Patients

Children attending the tertiary audiovestibular medicine outpatients in Alder Hey Children's Hospital between February 2016 and July 2019 were studied by a retrospective case note analysis. The research was conducted according to the rules and regulations of the Helsinki declaration relating to research involving live human subjects. The Health Research Authority of England (HRA) and Health and Care Research Wales (HCRW) approved the research (approval number 20/HRA/1289). The HRA also granted a consent waiver. Children with isolated findings of rare third windows were included. We defined these as rare third window disorders as they are the least reported or not reported at all and thus they did not include EVA or SSCD. Children who were diagnosed with a systemic genetic syndrome with third window structural abnormalities and cochleovestibular dysplasias of varying nature were also excluded. The age range for the study was fixed between 5 and 17 years as bony structural abnormalities like SSCD could be a part of normal development up to the age of 5 years (10, 13, 24).

### Methods

#### Anamnesis

History from patients with third window disorders is crucial to establish a diagnosis. There are characteristic symptoms of the third window effect. However, in children, these may be difficult to elicit and, indeed, obtaining this history is an art in itself driven by several behavioral factors in the child (32). Thus, eliciting this history is often surrogate and dependant on carers or parents who usually are quite reliable and astute to observe hearing and vestibular behavior in the children. A lack of school performance and academia or behavioral reactions to communication was deemed as key indicators of a hearing loss. Sudden falls and trips, lack of spatial awareness, bumping into objects, or inability to ride a bike were taken as indicators of disequilibrium. Wherever possible, children were asked about specific third window symptoms as older children were in a position to narrate these symptoms themselves. A full set of symptoms is shown in Table 2.

**Table 2:** Symptoms of pediatric vestibular disease [table adapted from (5)]; children with specific symptoms in the series in brackets and italics.

• Obvious dizziness/vertigo/lightheadedness (usually describable by children above 8 years of age) • Fright or pallor • Clutching at objects to steady oneself • Bumping into things, falling and tripping [*case 1*] • Clumsiness • Sudden very brief lasting falls with immediate complete recovery • Periodic episodes of nausea or vomiting ± migrainous features • Delayed motor functions • Loss of postural control or unsteadiness [*cases 2,3,6*] • Difficulty with ambulating in the dark [*case 2*] • Difficulty with or avoidance to ride a bike or in amusement park rides due to imbalance [*cases 2 and 4*] • Abnormal movements during walking, running [*case 7*] • Abnormal behavior observed up by significant others (care giver, school or peer group) • Difficulties in challenging movements (swimming, dancing) • Oscillopsia • Difficulties in challenging visual environments for example in superstores and in crowded places [*cases 2 and 3*] • Poor head eye or hand eye coordination • Motion intolerance or cyclical vomiting [*case 2*] • Third window symptoms if described by older children—conductive dysacusis (for example, hearing one's own footsteps), gaze evoked tinnitus (audible eye movements [*case 3*]), autophony (altered perception or perverted self-monitoring of own voice [*case 3*]), Tullio's phenomenon (dizziness on hearing loud sounds), Hennebert's phenomenon (pressure induced dizziness for example on coughing and sneezing), pulsatile tinnitus (tinnitus that is synchronous with pulse beat [*cases 3 and 5*])

#### Audiovestibular Quantification

All children and carers provided full verbal informed consent for clinical examination. The examination was performed by the first three authors, all of whom are experienced clinicians. Complete pediatric examination is an essential part of the holistic assessment of the child, and indeed problems with communication or with balance may result from non-audiovestibular conditions, and the possibilities are vast. A full neurological, oculomotor, and musculoskeletal examination were performed in every child, especially as disequilibrium may be a presenting feature of a neurological, ocular, or musculoskeletal disorder.

Audiological tests performed in every child included behavioral pure tone audiometry and live voice speech tests as well as objective audiometry with tympanometry, acoustic reflexes (ART), and transient otoacoustic emissions. Otoscopy was performed before audiological testing. Pure tone audiometry entailed measurement of air and bone conduction thresholds with masking wherever indicated with the sound delivered through TDH 39 headphones. Up to 20 dBHL thresholds were considered as normal, and a negative bone conduction was indicated by a threshold of below 0 dBHL. Pure tone thresholds were measured from 500 Hz to 4 kHz and were averaged for the study. Transient otoacoustic emissions (TEOAE) were measured by Otodynamics equipment with a stimulus intensity of 80–88 dBSPL.

A full neurovestibular examination was performed first with vision. This included measurement of the subjective visual vertical (measurement of head tilt with respect to the vertical to assess static gravitational sensor function) and any nystagmus with optic fixation (for central function). Videonystagmography (VNG) using the ICS system with and without optic fixation was used to measure smooth pursuits and saccades (for central function), nystagmus (for peripheral vestibular semicircular canal and central function), post passive head shake nystagmus in the horizontal direction (for peripheral lateral semicircular canal function), and in the vertical direction (for central function), the mastoid vibration test induced nystagmus (for peripheral lateral semicicular canal function), the head heave test (otolith counterpart of the high frequency canal head impulse test to assess high frequency utricular function), the ocular counter rolling test (ocular movements in response to head roll to assess gravitational sensor function), the office rotatory chair tests (to assess peripheral vestibulo-ocular reflex or VOR), the optokinetic test (for central function), and the suppression of visual fixation test (for central cerebellar function). Vestibulo-spinal tests were performed with the Romberg, the Unterberger, the tandem gait, the one legged stance, and the sharpened Romberg's tests. A foam cushion was used to eliminate proprioception cues in these tests with eyes closed to elicit a vestibular response in maintaining posture. Dix Hallpike, the supine roll test, and the deep head hanging test were performed to exclude benign positional paroxysmal vertigo (BPPV).

A full 6 canal video head impulse test (vHIT) was performed in every child with a minimum of 10 head thrusts for each canal function with the ICS Impulse system. A VOR gain of 0.8–1 was considered normal for the lateral semicircular canal whilst a VOR gain of 0.6–0.8 was the norm for the vertical canals. Recent studies (33, 34) indicate that vertical canal gains are lower in the pediatric population than in adults similar to what we have also found using similar equipment in children with normal vestibular function (35). Saccades, rather than VOR gain, were deemed as pathological weakness as studies have shown that saccades can occur with normal VOR gain in vestibular hypofunction (36, 37). Two senior clinicians (SDG and SR) analyzed the saccades in the current series independently. Low VOR gain without saccades was deemed as clinically insignificant in the absence of any neurological comorbidity. Compatibility with the test was high. Calorics are not performed in our center due to the distress they cause to children.

Cervical vestibular evoked myogenic potential test (cVEMP) was performed with the Neurosoft software. Air conducted stimuli delivered through Etymotic ER 3A insert ear phones at rarefied 100 dBnHL comprised of 60 sweeps with a stimulation rate of 3–5 Hz were presented to each ear at a tone burst of 500 Hz with a Bartlett Trapezoid rise and fall time of 1 ms. The analysis time window was 50 ms with a sampling rate of 5,000 Hz. Amplitudes were measured after averaging at least 2 runs wherever possible. Adaptive notch filter between 30 and 2,000 Hz was used in the protocol. Rectified amplitudes were also considered when subsequent measurements of asymmetry were performed between the 2 sides. The amplitude asymmetry was calculated as the right amplitude minus left amplitude divided by right amplitude plus left amplitude × 100. Thresholds were measured wherever possible, but it must be remembered that this is not always possible due to compatibility issues as active sternocleidomastoid contraction becomes strenuous for some children and several runs cannot be implemented. Pediatric norms are variable and the test itself is very operator dependant. The normative value the (38) first paper of its kind as regards mean amplitudes and thresholds in children were different from other publications (39, 40). It was also emphasized that this variability is due to several factors that include VEMP stimulus parameters and local laboratory norms (39). Absolute amplitude values may be misleading due to the lack of these standardized norms and hence we follow the asymmetrical amplitude parameter as a more robust sign than absolute amplitudes, unless these amplitudes are clearly very high and match with symptoms. In our center, we consider 15–150 microvolts as normal amplitude values, up to 25% as normal amplitude asymmetry and 85 dBnHL as the threshold with our test set up in the pediatric population. We are still collecting our own ocular vestibular evoked myogenic potential (oVEMP) norms, so we did not use this test in this series. Interestingly, in a recent study, the authors commented that oVEMPs are more sensitive indicators than cVEMPS to diagnose vestibular dysfunction in children (41).

All children presenting with hearing loss underwent the full set of aetiological investigations as suggested by the British Association of Audiovestibular Physicians (BAAP) (42) that included chromosome karyotyping, molecular biology genetic studies, ophthalmological investigations, and metabolic and inflammatory screens to rule out other causes of hearing loss in children. Some of these tests were also informative of causes of vestibular dysfunction in children, e.g., autoimmune vestibular disorder.

It must be emphasized that pediatric examination and audiovestibular testing is intense and time consuming. Occasionally, the children were brought back for a second appointment. Every effort was made to make the child as comfortable as possible and not put too much strain as, in our experience, tiredness and fatigue during testing invariably leads to less test compatibility with the tests generating incomplete results. This situation is hardly encountered in adults.

Table 3 shows the examination algorithm.

**Table 3:** Assessment of the vestibular system in children [table adapted from (5)].

I. Audiological tests • Pure tone audiometry with masking • Tympanometry • Acoustic reflexes • Otoscopy • Transient otoacoustic emissions II. Full neurological examination III. Musculoskeletal examination IV. Full oculomotor examination V. Vestibular tests • Assessment of subjective visual vertical • Videonystagmography with and without visual fixation for smooth pursuits, saccades, horizontal and vertical head shake, head heave, ocular counter rolling, mastoid vibration test, optokinetic test and ectopic eye movements • Video head impulse test • Cervical vestibular evoked myogenic potential test • Vestibulo-spinal test battery with and without proprioception for Romberg, Unterberger, tandem gait; one legged stance and sharpened Romberg • Office rotatory chair tests and suppression of visual fixation • Dix Hallpike, supine roll and deep head hanging tests

#### Imaging

Based on the history, clinical examination, and investigations, all children with conductive/mixed hearing losses and normal middle ear function with/without balance problems, third window symptoms, and deranged vestibular function tests underwent HRCT to visualize the bony otic capsule as a first line of investigations. Only sensorineural hearing losses also underwent HRCT if their MRI scans were deemed normal. The CT was acquired using ultrahigh resolution spiral CT with overlapping slices of 0.8 mm with 0.4 mm increment. The images were reconstructed in the axial, coronal, and sagittal planes with oblique views at 0.5 mm. The scans were analyzed by one of the co-authors (SA) who is a senior radiologist specializing in pediatric head and neck radiology. The thickness of the semicircular canal walls was measured and a thickness of at or <0.5 mm in at least 2 planes was deemed as a thin semicircular wall or a near dehiscence (26, 43). HRCT provided the final direct visual confirmation of the rare third windows in the children in the series.

### Statistical Methods

Descriptive statistics were computed using Quick Statistics Calculators, an online digital portal (https://www.socscistatistics.com/tests). We did not investigate any analytical statistics to explore variations among groups as the number of cases were deemed too small, and there is a danger of running ANOVA with small samples in that it might lead to erroneous conclusions because of a lack of power (44).

## Results

**Figure 1:**
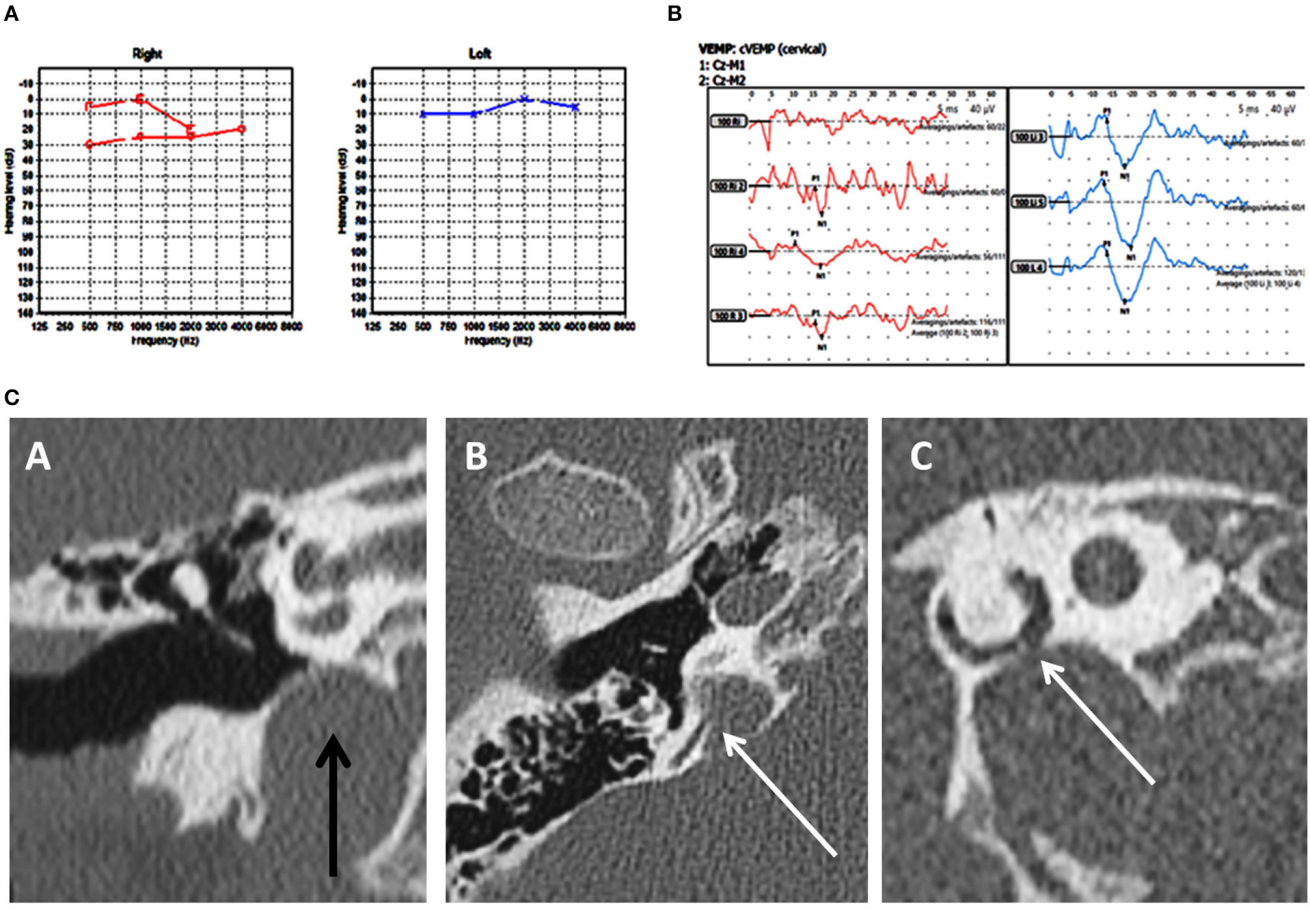
Case number 2—Unilateral right posterior semicircular dehiscence. **(A)**, Pure tone audiometry showing mild conductive low frequency hearing loss on the right. **(B)**, Clinically significant cVEMP amplitude asymmetry with the right weaker than the left. This child perceived significant balance issues but his vestibular tests including the vHIT was normal. **(C)**—CT scan images of the right petrous temporal bone in the coronal plane (A) demonstrates a high riding jugular bulb. The axial image (B) the sagittal oblique reconstruction parallel to the posterior semi-circular canal (C) demonstrate dehiscence of the posterior semi-circular canal at its junction with the jugular bulb.

**Figure 2:**
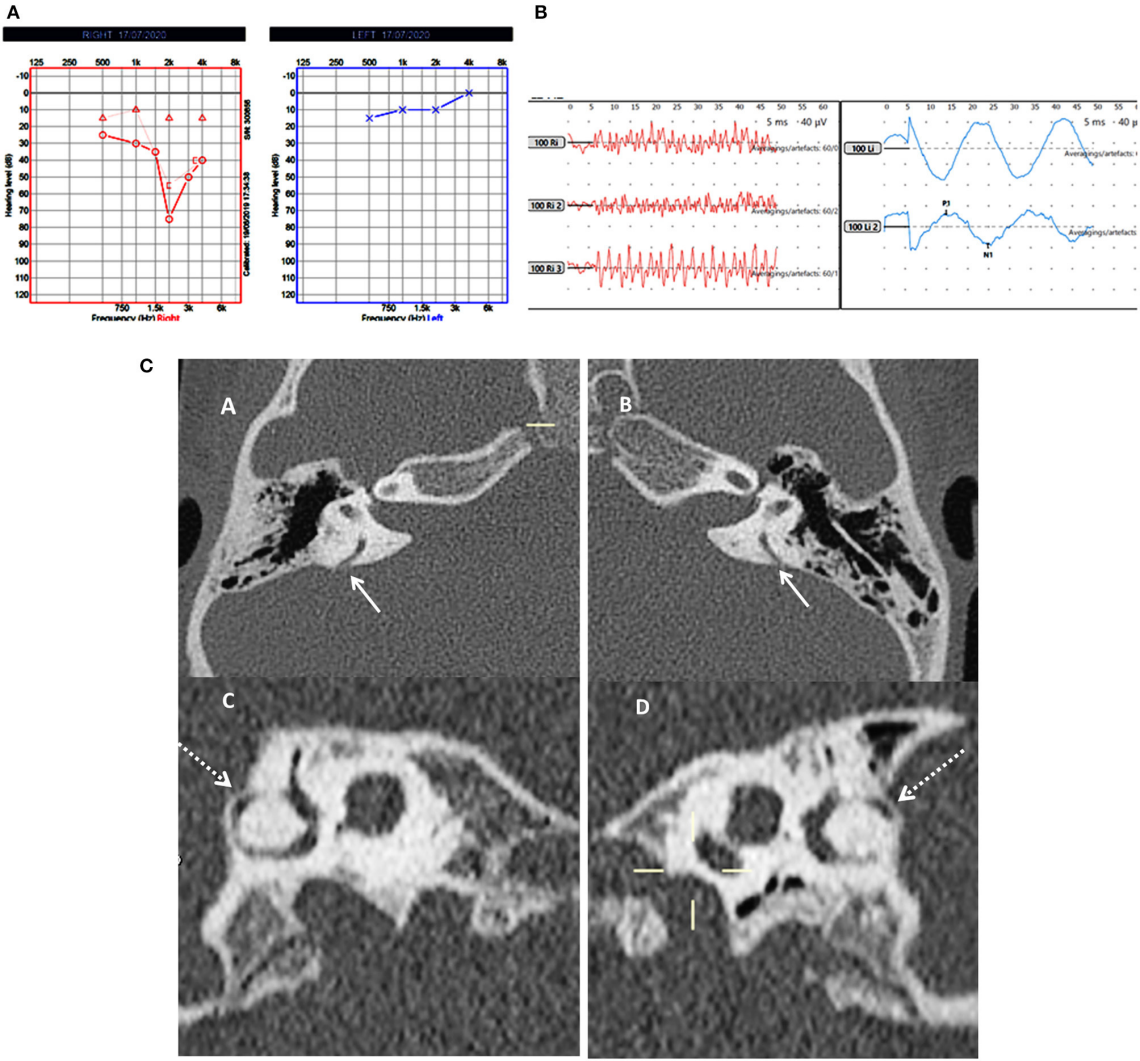
Case number 5—Bilateral posterior semicircular canal thinning. **(A)**, Pure tone audiometry showing right mixed hearing loss. **(B)**, cVEMP showing absent response on the right and normal amplitude on the left; in this child vHIT was normal and there were no symptoms of balance problems. **(C)**—CT scan images of the right (A) and left (B) petrous temporal bone in the axial plane demonstrates apparent dehiscence of the posterior semi-circular canal (white arrows). Sagittal oblique reconstruction of the right (C) and left (D) petrous temporal bone parallel to the plane of the posterior semi-circular canal demonstrates thinning of the overlying bone (dotted arrows) measuring 0.5 mm in thickness on both sides.

**Figure 3:**
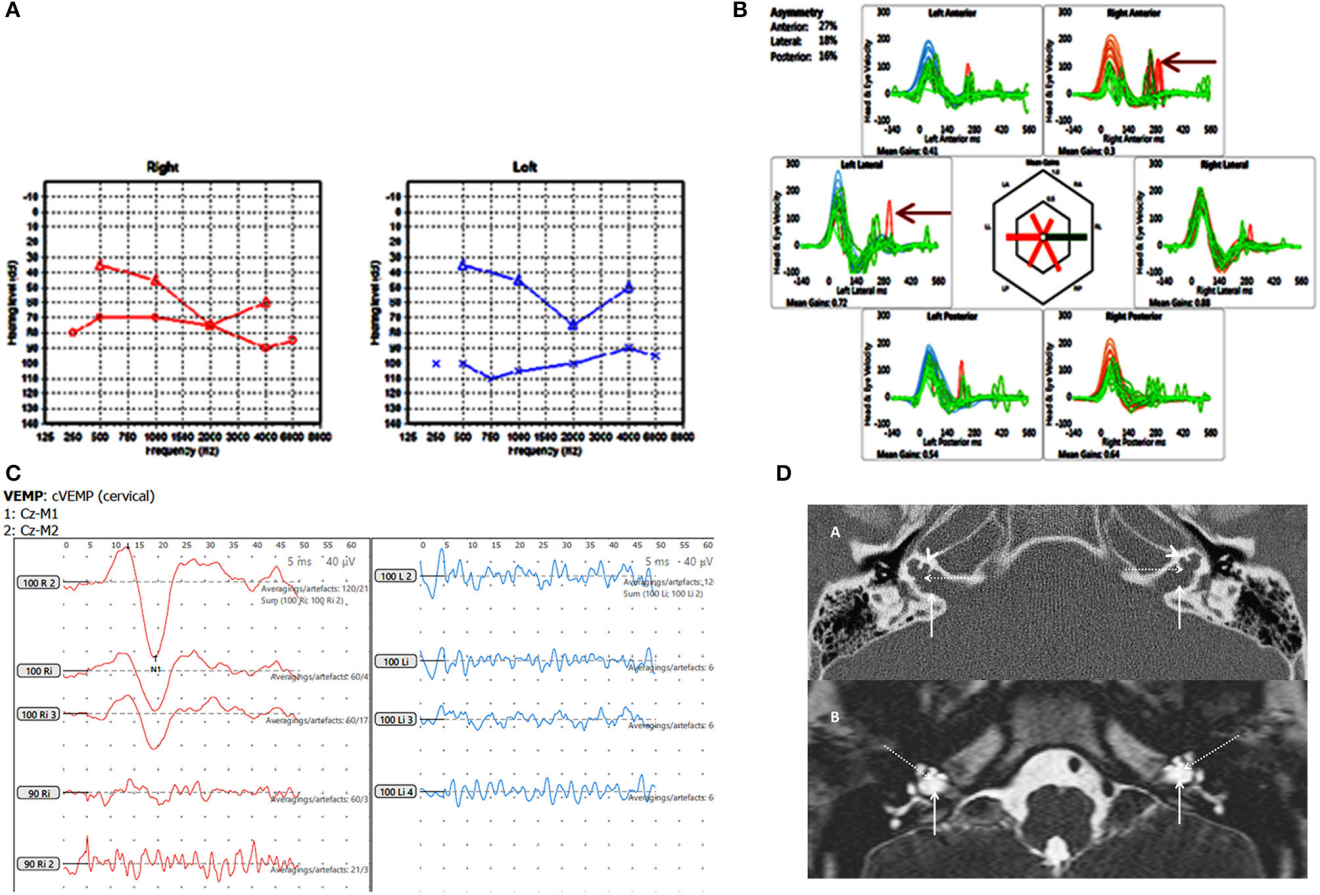
Case number 6, X linked gusher. **(A)**, Pure tone audiometry showing significant bilateral mixed hearing loss. **(B)**, Catch up saccades on both sides on the vHIT (arrow). **(C)**, Absent cVEMP response on the side of the greater hearing loss and normal amplitude and threshold on the other side. **(D)**, Axial CT scan (A) and T2 DRIVE MRI image (B) of the petrous temporal bone demonstrating bilateral bulbous dilatation involving the fundus of the internal auditory canal (arrow) and bilateral incomplete separation of the basal turn of the cochlea (arrow head) from the fundus of the internal acoustic canal (dotted arrow) classical of X linked gusher disorder.

**Figure 4:**
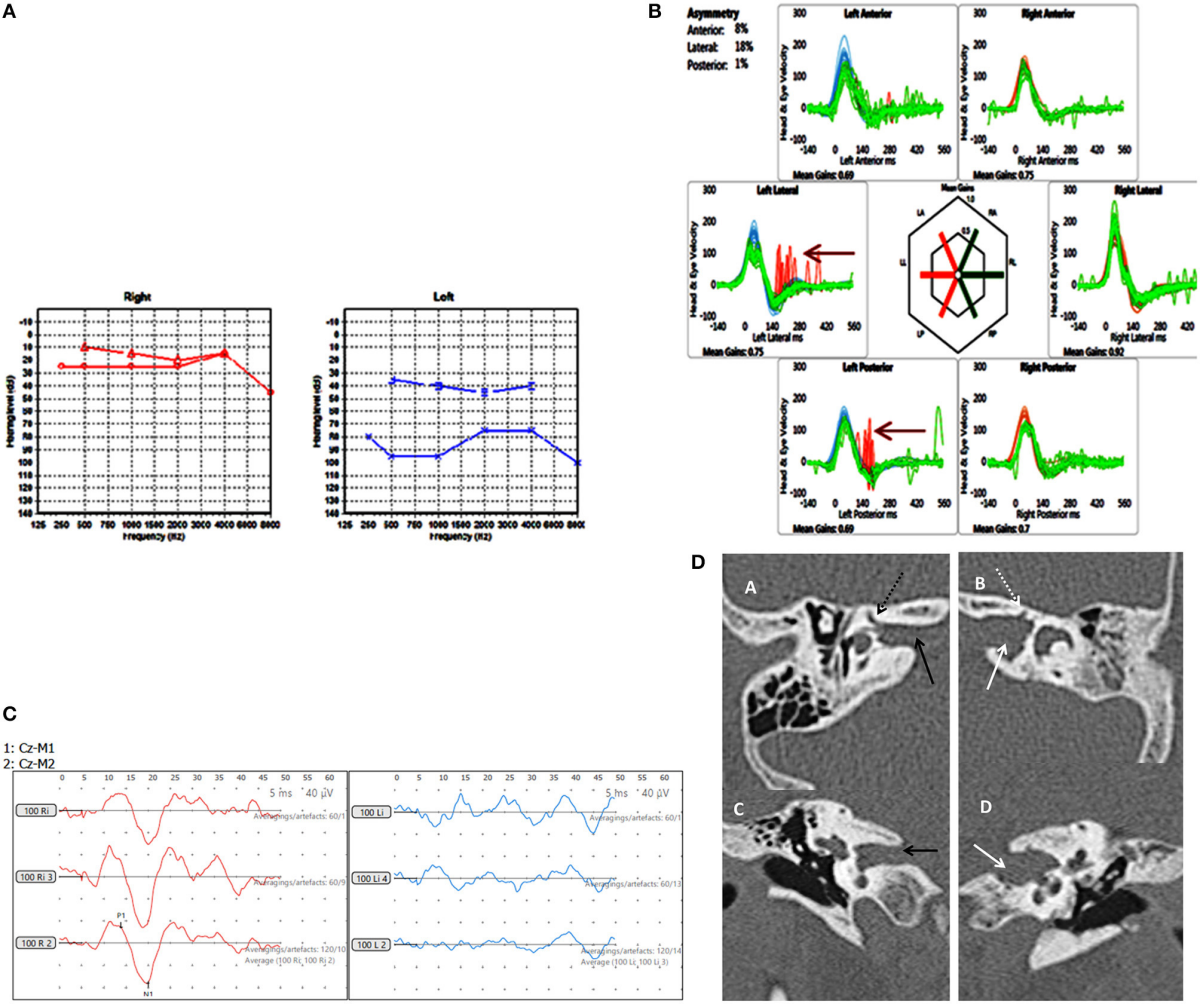
Case number 8, Multiple abnormalities. **(A)**, Pure tone audiometry showing left mixed hearing loss (A) with a normal right side. **(B)**, vHIT showing left sided high frequency multiple canal dysfunction with catch up saccades (arrow) with a normal right side. **(C)**, No response on the cVEMP on the left with a normal right side. **(D)**, CT scan of the left petrous temporal bone in the axial plane (B) and coronal plane (D) demonstrate irregular widening of the internal auditory canal (white arrow) the labyrinthine segment of the left facial nerve canal has an ill-defined bony wall (dotted white arrow). The right petrous temporal bone demonstrates a normal sized internal auditory canal (C black arrow) on the axial and coronal planes and the bony wall of the right facial nerve canal appears normal (A dotted black arrow).

The observations in the case series are given in Tables 4, 5, Figures 1–4 are representative cases in each group (Group A PSCD—Figure 1; Group B posterior semiciruclar canal thinning PSCT—Figure 2; Group C X linked—Figure 3, and Group D Multiple—Figure 4).

**Table 4:** Children in case group.

**Child/group**	**HS**	**BS**	**TW**	**Tymp/ECV**	**ART**	**OAE**	**VNG/VFT**	**PTAav RAC/BC**	**PTAav LAC/BC**	**Type HL**	**Diagnosis**
1/A	Yes	Yes	Nil	Normal	Normal	Absent	Normal	52/46	55/46	Mix B	R PSCD
2/A	Nil	Yes	Nil	Normal	Absent R	N/A	Normal	25/5	6/0	CHL R	R PSCD
3/A	Nil	Yes	Auto/GET/PT	Normal	Normal	Normal	Normal	7/4	5/3	No HL	R PSCD
4/B	Yes	Yes	Nil	Normal	Normal	Normal	Abnormal	36/30	37/26	Mix B	R PSCT
5/B	Yes	Nil	PT	Normal	Normal	Normal	Normal	42.5/30	4/0	Mix R	Bil PSCT
6/C	Yes	Yes	Nil	Normal	Absent L	Absent	Normal	76/51	100/51	Mix B	X linked
7/C	Yes	Yes	CD	Normal	Normal	Absent	Abnormal	80/50	80/50	Mix B	X linked
8/D	Nil	Nil	Nil	Normal	Normal	Absent L	Abnormal	25/10	100/53	Mix L	Multiple L
**MeanPTAthresholds**								42.93/ 28.25	48.37/ 28.65		

*HS, hearing symptoms; BS, balance symptoms; TW, Third window symptoms; Tymp, tympanometry; ECV, external auditory canal volume; ART, acoustic reflex test; OAE, transient otoacoustic emission; VNG, videonystagmography; VFT, vestibular function tests; PTA av R and av L, pure tone audiometry thresholds averaged 500 Hz−4 kHz right and left in dBHL; AC, air conduction; BC, bone conduction; HL, hearing loss; CHL, conductive hearing loss; Mix, mixed; Bil/B, bilateral; R, right; L, left; PSCD, posterior semicircular canal dehiscence; PSCT, posterior semicircular canal thinning; N/A, not available; PT, pulsatile tinnitus; GET, gaze evoked tinnitus; Auto, autophony; CD, conductive dysacusis*.

**Table 5:** Children in case group, vHIT and cVEMP results.

**Child/group**	**VOR L LSCC**	**VOR R LSCC**	**VOR L SSCC**	**VOR R SSCC**	**VOR L PSCC**	**VOR R PSCC**	**Saccades**	**cVEMP amp/RA R μV**	**cVEMP amp/RA L μV**	**Thresh R/L dBnHL**
1/A RPSCD	1.16	1.09	0.55	0.75	0.85	0.69	Yes R	254.9/2.9	168.8/2.2	85/85
2/A RPSCD	0.93	0.89	0.56	0.67	0.84	0.54	Nil	42.8/0.9	114.2/2	NA
3/A RPSCD	0.67	0.48	0.62	N/A	N/A	1.46	Nil	NA	NA	NA
4/B RPSCT	0.86	0.9	0.73	0.48	0.54	0.47	Yes R	Nil	Nil	Nil
5/B BPSCT	0.83	0.88	0.58	0.42	0.55	0.56	Nil	Nil	57.7/1.7	Nil/NA
6/C Xlinked	0.72	0.88	0.3	0.41	0.54	0.64	Yes B	92.5/2.9	Nil	85/Nil
7/C Xlinked	1.07	0.95	0.69	0.8	0.95	0.69	Nil	57.3/1.1	58.9/0.8	75/75
8/D LMulti	0.75	0.92	0.69	0.8	0.95	0.69	Yes L	101.2/1.5	Nil	NP/Nil
**Mean VOR gain**	**0.87**	**0.88**	**0.59**	**0.61**	**0.7**	**0.71**				

*VOR, vestibulo-ocular reflex gain; LSCC, lateral semicircular canal; SSCC, superior semicircular canal; PSCC, posterior semicircular canal; amp, amplitude; RA, rectified amplitude; Thresh, threshold; R, right; L, left; NA, not available; PSCD, posterior semicircular canal dehiscence; PSCT, posterior semicircular canal thinning; B, bilateral; Multi, multiple; NA, not available; NP, not performed; Nil, absent response*.

The total number of children seen for vestibular assessment between the period of February 2016 and July 2019 were 920. Out of these, 19 were diagnosed with SSCD (2.06%), 26 with EVA (2.82%), and 8 with rare third window disorders (0.86%) on HRCT. These rare third window disorders included 3 with isolated posterior semicircular dehiscences (0.32%—cases 1,2,3), 2 with thinned posterior semicircular canal wall (0.2%—cases 4,5), 2 with X linked gusher (0.2%—cases 6,7), and 1 with a combination of a facial nerve canal hypoplasia and a dilated auditory meatus lying very close to the cochlea (0.1%—case 8). The diagnosis of the X linked gusher group was by typical HRCT findings and a typing of the POU3F4 genetic mutation in a family of 2 children with the same mother. Two of the 3 children with frank PSCD also showed high riding jugular bulbs. These children where rare third window disorders were identified were assigned 4 groups: Group A—children with only posterior semicircular canal dehiscence (PSCD); Group B—children with a thinned posterior semicircular canal wall (PSCT); Group C—children with X linked gusher disease; and Group D—children with other rare third window disorders.

Of the whole third window cohort (*n* = 53) that constituted only 5.76% of all children seen, Group B and Group C were observed in 3.77%, Group A in 5.66%, and Group D in 1.88%. There were 4 females and 4 males in the rare third window series (*n* = 8). The average age of the females was 11.75 years (range 6–15 years) and that of the males was 10.75 years (range 6–16 years). Of the 16 ears studied, a third window abnormality was observed in 4 ears on the right, 1 on the left, and in 3 children, it was present bilaterally with 5 ears showing no abnormality.

Children presenting with symptoms of communication difficulties, loss of hearing, and difficulties in understanding speech and instructions in the school set up were observed in 5 children (62.5%) of the cohort of rare third window disorders. There were 3 children who did not present with any symptoms of hearing loss, 2 of them with unilateral hearing losses, and 1 with normal hearing. Six children demonstrated a mixed hearing loss (75%) with appreciable air bone gaps in pure tone audiometry. There was 1 child with a conductive hearing loss only. There were 4 bilateral and 3 unilateral hearing losses. Average air conduction thresholds (the mean of the summated averages of air conduction thresholds in each child between 500 Hz and 4 kHz) and average bone conduction thresholds (the mean of the summated averages of bone conduction thresholds in each child) indicated a >10 dBHL air bone gap (Table 4). The hearing loss localized to the side of the lesion in 7 children; in 2 children it was also present in the ear without a third window abnormality, and in 1 child it was observed only in 1 ear where there was a bilateral third window abnormality. The child with normal hearing showed a unilateral pathology. In Group B, this asymmetry was most noticed where the hearing loss was present in the ear without a third window and absent in the ear with a third window. Only 2 children, one in Group A and one in Group B presented with a third window symptom of pulsatile tinnitus, and the child in Group A also complained of gaze evoked tinnitus and autophony.

The most severe mixed hearing loss was detected in Group C, the X linked gusher group where it was bilateral, and in Group D with multiple third windows with a severe mixed hearing loss on the affected side. The least intense hearing loss was in Group B with PSCT group. As regards bone conduction thresholds, none of the children demonstrated a negative bone conduction threshold.

All children (*n* = 8) in the series demonstrated normal otoscopy and normal tympanometry with normal external auditory canal volumes (100%). Six exhibited normal acoustic reflexes—ART; there were 2 children who showed absent reflexes. Three returned normal transient otoacoustic emissions (TEOAE), and 4 showed absent emissions (3 bilateral and 1 unilateral). No data was available for one child.

Six children (75%) in the series presented with one or more features of disequilibrium as enumerated in Table 2. The child with multiple third window abnormalities did not complain of any symptoms relating to balance, and neither did the child with the bilateral PSCT. From the group perspective, 100% presented with the symptom(s) in Groups A and C and 50% in Group B. Three children were observed to exhibit abnormal balance function tests excluding the vHIT and VEMP (37.5%). In the vHIT test, 4 (50%) children demonstrated repeatable catch up saccades (i.e., saccades that were consistent and replicable) in at least 1 or more canals with or without normal VOR gain. In all these children the saccades localized to the side of the third window abnormality. This abnormality was detected in 1 child in each group. The average VOR gain in the whole series in the lateral semicircular canal as given in Table 5 was within the normal range of our laboratory.

cVEMPs could be performed in 7 children. One child in Group A did not undergo the test as we did not possess the facility at the time of diagnosis and the child's subsequent discharge to the adult services. Two children found it too strenuous to complete the threshold test and we could only obtain amplitudes here (cases 2 and 5). In 1 child in Group B, we could only perform one run to obtain amplitudes (case 5, Figure 2B), and 1 child did not have thresholds performed on the good ear (case 8, Figure 4C). The results were rather heterogeneous to average in this study, but overall cVEMP abnormalities were observed in all 7 children. These included increased amplitude on the dehisced side (Group A, case 1); amplitude asymmetry (Group A, case 2); absent response (Group B, cases 4, and 5; Group C, case 6; Group D, case 8), and low thresholds (Group C, case 7). This is given in Table 5.

## Discussion

In the current study we concentrated on rare third window disorders in children, the definition of which we have described in our Methods section. We observed such abnormalities in 0.8% in a large cohort of children, accounting for only 15.09% of all third windows, making these defects rare.

Whilst in adults and in children, it has been established that frank dehiscences of semicircular canal walls, either superior or posterior, are responsible for the phenotype, yet there are patients where a thinning of the canal walls or near dehiscence may lead to similar symptoms (28). These patients often respond to surgical management of semicircular canal dehiscences. Judging as to what is a thinning can be subjective, and there is no consensus as yet as to what are the physical dimensions of such thinning. For example, Ward (45) considers thinning as a thin strip of bone in their study with adults, whilst Kaur (43) after measuring actual semicircular canal bone thickness observed that thickness ranged from 0.4 to 2.08 mm with an average of about 1.5 mm. Meicklejohn (26) in children above the age of 4 years reported similar observations. Saxby (13) commented that thinning can be developmental but can lead to a dehiscence in the future. Based on these studies, for this study, we postulated that a semicircular canal wall thickness at or below 0.5 mm can be accepted as thinning.

Group A in our study comprised of frank posterior semicircular canal dehiscence as a single inner ear abnormality. This has been hardly reported in children. Meicklejohn (26) in his large series studying CT temporal bones in children from birth did not find any PSCD between the ages of 4–7. In another large series studying temporal bones, PSCD was observed only in 0.6% of children above 3 years (13). In the only case series investigating PSCD in children, 3 children were studied who presented with unilateral PSCD (46). The current series showed a slightly lesser incidence than the one reported in Meicklejohn's (26) series and in Saxby's (13) series due to the fact that these studies included concomitant cochleovestibular dysmorphia that we have excluded from our study. Two out of our three children in Group A also had a high riding jugular bulb that is deemed as an association of PSCD (8, 13, 46, 47) and in both of these children, the point of dehiscence was in contact with the jugular bulb (Figure 1C).

Clinical features of PSCD can be variable. In the only pediatric clinical series comprised of 3 children, normal hearing was reported in addition to low frequency conductive hearing loss. They all presented with third window symptoms and all showed cVEMP abnormalities with increased amplitudes and decreased thresholds (46). In the current series, we observed some heterogeneity of symptoms. Mixed hearing loss, conductive hearing loss, and normal hearing were observed. All the children presented with disequilibrium. Rather interestingly, the child with right PSCD perceived quite disproportionate balance problems and showed a significant VEMP amplitude asymmetry with the right side weaker than the left. One reason for this might be due to intrinsic saccular weakness in this child that explains the child's disproportionate balance symptoms. In the child with unilateral left PSCD, bilateral mixed hearing loss, abnormal vHIT, and high VEMP amplitudes, it is possible that a structural third window may be evolving, a proposition suggested by Saxby (13). The third child in this group presented with some typical third window features.

Group B in our study consisted of children with PSCT with audiological and balance symptoms that to our knowledge have not been reported in literature. This group was rather homogeneous in terms of their audiovestibular phenotype and yielded some consistency in their VEMP results. They showed absent either unilateral or bilateral VEMP responses. The series with PSCT in adults also observed that about 30% of their subjects did not return a VEMP response (45). Again we propose that this could be due to inherent saccular weakness in this condition. Both children showed bilateral mixed losses with normal OAE and ART.

Group C in our study were the 2 children with a congenital X linked gusher. They both showed identical pathognomonic HRCT features that is usually diagnostic (48). They both presented with bilateral severe mixed hearing losses and balance problems. Both showed a cochlear component to the hearing loss. One child fulfilled the criteria for a third window disorder with lowered VEMP thresholds whilst the other with an abnormal bilateral vHIT showed an absent VEMP response on the side of the greater hearing loss. This may suggest that both cochlear and saccular function can be affected in this disorder.

The third window effect in an X linked gusher is postulated to be due to the absence of lamina cribrosa establishing an abnormal connection between the perilymphatic space and the subarachnoid space, i.e., a connection between the inner ear and the cranial spaces (8). The condition is rare. A thorough search of the literature yielded circa 89 patients since 1971 (29, 47, 49–58). These children present with progressive mixed hearing losses and varying degrees of vestibular problems as was found in our study. A dilated internal auditory meatus (IAM) in these children accompanied the inner ear phenotype as such dilatations frequently accompany inner ear dysmorphology (59, 60). This is the first time that we are presenting objective quantification of vestibular function in X linked gusher.

Our child in Group D was rather interesting. The severe left sided mixed hearing loss with normal middle ear function suggested inner ear abnormalities with third window structural defects as these are the only pathologies known to generate a non-middle ear origin air bone gap (8). Therefore, we deduced that the conductive element of the mixed hearing loss can come only from a third window defect. The CT showed multiple third window structural abnormalities. We included this child to highlight the observation that occasionally known third window structural abnormalities might not show up clearly on imaging but can be inferred by the effects they generate.

The child in Group D clearly showed deficient vestibular function on the left side, suggesting a cochleo-vestibular pathology. This child was also the one who showed no symptoms from the audiovestibular function point of view. A child may undergo complete vestibular central compensation rendering the child asymptomatic (35) and might not perceive a unilateral hearing loss (61).

About two thirds of children in the present series did not complain of a subjective hearing loss, including children who demonstrated PTA measured mild hearing loss, a unilateral hearing loss, or normal hearing. The majority of children in the current series showed a mixed hearing loss or a conductive hearing loss that is in agreement with other studies who have described similar hearing loss in SSCD in children and third windows (20, 23, 25, 62). The hearing loss correlated well to the side of the lesion in the majority. Negative BC has been postulated to be a diagnostic criteria for third window defects especially SSCD (9). However, Merchant et al. (63) commented that rather than negative BC thresholds, the air bone gap is more important to consider as a diagnostic criteria. In the current series, there were no children with negative BC.

Normal TEOAE was observed in 3 children (1 with normal hearing and 2 with 30 dBHL or less hearing loss). TEOAE are abnormal in hearing losses of cochlear origin above 30 dBHL (64), so probably these 2 children had a mild cochlear component to their hearing loss. TEOAEs are usually preserved in third window disorders unless complicated by a simultaneous significant cochlear pathology that over rides the third window effect (5, 46, 65). The children with mixed losses above 30 dBHL in the series returned absent TEOAEs. Sensorineural hearing loss has been reported in pediatric SCDS (5, 66–68). There was 1 child with normal hearing that has also been reported in third window disorders (5, 68).

Tympanometry and ART are also preserved in third window disorders (69). In our study, tympanometry was normal in all children that virtually eliminated a middle ear disorder explaining a mixed or a conductive hearing loss. ART was present in three-fourths of cases. The sensitivity and specificity of the test is not 100% and we would consider its absence in one-fourths of the cases as a normal variation (70).

In the current series, three-fourths of the children in the series complained of some features of disequilibrium that is characteristic of a third window abnormality (47). However, balance symptoms may be absent altogether (5, 20, 47, 71). We believe that this could be due to central compensation. There were only 2 children who presented with classical third window symptoms in the form of pulsatile tinnitus, gaze evoked tinnitus, and autophony. Dasgupta and Ratnayake (5) in a series with SSCD in children remarked that radiologically established pediatric third window disorders in children might not present with a fully blown clinical syndrome with its classical features as this history may be difficult to elicit or the defect has still not reached the stage where it may lead to classical third window symptoms.

Vestibular function tests other than the vHIT and cVEMP were normal in about 60% of our children but abnormal in about 40%. They were mostly abnormal in children with a possible cochlear abnormality. Vestibular function test except the VEMPs results are variable in third window disorders (8, 47) but curiously they have not been studied in detail. We believe that these tests are more likely to be deranged if the third window abnormality involves a wider anatomical topography of the bony labyrinth.

The vHIT as a tool to assess high frequency all canal function in vestibular diagnostics has revolutionized the diagnostic process and finds wide application (72). Use in children is still limited although the evidence is slowly emerging (35, 73–78). One difficulty in children is the standardization of norms. We have explained about these norms in our Methods section (33–35). The average VOR gain in our series in all canals was mostly normal in the 4 different groups that we have also found in a previous study (5) suggesting that VOR gain is largely preserved in pediatric third window disorders.

The role of saccades in interpreting the vHIT has gathered momentum (79) and we have explained the importance of saccades in the presence of normal VOR gain in our Methods section (36, 37). In the current series, they were deemed pathological in about a half of the cohort localizing accurately to the side of the third window defect. One publication (5) reported the utility of the vHIT in pediatric SSCD, and it appears from this study that indeed this test does add significantly to vestibular information in third windows. For example, in EVA, vHIT can be deranged (80).

VEMP studies have shown that third window VEMP characteristics [i.e., increased amplitude and decreased threshold due to hypersensitivity of the saccule and the urticle to acoustic energy (19)] may be observed in third window disorders that include EVA, SSCD, PSCD, and CFD in the pediatric population (3, 46, 81, 82). We have discussed the variable norms for pediatric cVEMP in our Methods section (38–40) and the difficulties in performing the test in children. For oVEMPS, there are very few studies (83, 84) to establish norms. In the current series, there were increased amplitudes, absent responses, and low thresholds in the children undergoing cVEMPs in varying percentages in the cohort. This indicates that cVEMPs characteristics in pediatric rare third windows may be rather heterogeneous. Overall, cVEMP abnormalities were detected in all the children who underwent the test. This can suggest that saccular abnormalities may be associated with a high percentage of rare third window disorders in children.

The limitations of this study include the small numbers, but the third window conditions highlighted in the current series are rare in children. Therefore, it will be injudicious to generalize observations based on this study. In addition, this was a retrospective non-controlled study. However, we were careful to avoid inconsistencies as the 2 senior and experienced physicians (SD and SR) managed these children maintaining continuity of observations, thereby eliminating an important bias in the study. Furthermore, the study looked into a defined set of the population unlikely to be influenced by confounding variables.

As the literature suggests, third window disorders in children may present without third window syndromic features (5) which are determined by defined symptoms and objective signs as is found in the literature mainly in adult cohorts (9). A number of factors may account for this, for example, a co-existing cochlear or vestibular dysfunction. It could also be due to a difference in endolymphatic fluid dynamics in children as compared to adults (85). Furthermore, as mentioned earlier, third window symptoms may be difficult to elicit in children. In children, consequently, the diagnosis is based on a holistic process rather than by didactic and set criteria of a third window syndrome so well-defined in adults. However, some features like disequilibrium, conductive component of a hearing loss with normal middle ear studies, and normal TEOAE may be consistent features that should raise the suspicion of a third window. There may be accompanying vestibular dysfunction and VEMP abnormalities.

This study has highlighted a cochlear element to a mixed loss in these rare third windows as a phenotype. It has also observed 2 new entities that present with features of third window disorders. The first one is a PSCT or near dehiscence that behaves like other third window disorders, and the second is a combination of more than 1 possible third window structural abnormality which are as yet unclassified third windows but generate symptoms nevertheless.

It is important to consider the premise that whether HRCT diagnosis of a third window abnormality, especially a canal dehiscence in a child, can be incidental or part of the normal developmental process and therefore deemed non-pathological. Did the HRCTs over diagnose the conditions in our series? Available evidence suggests that dehiscences can be a normal phenomenon until the age of 5 years (13, 26). All our patients were over 5 years, and we feel that an incidental or developmental third window structural defect is unlikely to be the case, as all these children in the series presented with at least one third window feature and were most comprehensively investigated from other causes of a hearing loss. Therefore, by the process of elimination in the medical algorithm, we concluded that their observed third window abnormalities were responsible for their phenotypes. Thus, it is a matter of fine judgement and expertise to diagnose these conditions in children. HRCT remains an important investigation to perform to establish diagnosis that aids significantly in informing the child and the carers as to what is going on and may determine surgery if required.

We believe that it is important to consider third window disorders as concrete diagnoses as this helps in formulating holistic management plans in children. In our center, all these children and their parents/carers receive full counseling on typical third window syndrome symptoms that can occur later in life. Indeed, this dissemination of diagnostic information often participates in a cognitive treatment of the child. One of the children in the current series with autophony and gaze evoked tinnitus who was desperately seeking answers (being labeled as someone having psychological problems with a poor quality of life) was extremely relieved with the diagnosis and devised excellent coping strategies by self-awareness. None of the children in our series required operative intervention, but some did require auditory, vestibular, and cognitive rehabilitation aided by the diagnostic process.

## Conclusions

Rare third window disorders, as the name suggests, are rare and can be missed unless there is a high index of clinical suspicion in a child with disequilibrium, a conductive element to a measured hearing loss with normal middle ear function and abnormal objective vestibulometry that will lead to a confirmation with HRCT. They might not present with classical third window symptoms described well in adults, and their phenotypes might be quite heterogeneous. This study shows that diagnosis of these conditions in children is dependent on a good anamnesis and extensive objective and subjective audiovestibulometry and depends on expert and fine clinical judgement. It also emphasizes that it is important to diagnose rare third window disorders in children for their holistic management.

## Data Availability Statement

All datasets generated for this study are included in the article/supplementary material.

## Ethics Statement

The studies involving human participants were reviewed and approved by HRA England and Health and Care Research Wales (HCRW); approval number 20/HRA/1289 dated 19.05.2020; parental/carer consent waived as this was a retrospective case note study. Written informed consent from the participants' legal guardian/next of kin was not required to participate in this study in accordance with the national legislation and the institutional requirements.

## Author Contributions

SD and SR were the responsible clinicians for the children included in the case series. SD collated the data, collected and analyzed the evidence, and wrote the paper. RC and SR collaborated to write the manuscript and analyse the evidence. JI and LS were the audiologists and SA was the radiologist responsible for the children in the case series. All authors critically reviewed clinical findings and the manuscript drafts, and contributed to the final version and own responsibility for the integrity of the paper.

## Conflict of Interest

The authors declare that the research was conducted in the absence of any commercial or financial relationships that could be construed as a potential conflict of interest.
